# Migrated Mesh Plug Masquerading as a Bladder Tumor

**DOI:** 10.1089/cren.2016.0125

**Published:** 2017-01-01

**Authors:** Claudia Sevilla, Daoud Dajani, Monish Aron

**Affiliations:** USC Institute of Urology, University of Southern California, Los Angeles, California.

**Keywords:** mesh erosion, robotic surgery, gross hematuria, bladder cancer

## Abstract

***Background:*** The purpose of this case presentation is to demonstrate how erosion of mesh into the bladder can initially present with the same symptoms as bladder malignancy.

***Case Presentation:*** A 62-year-old Hispanic male presented with 2 years of hematuria along with imaging concerning for a bladder tumor. The patient underwent cystoscopy with biopsy of a lesion at the anterior bladder. It was ultimately determined that a mesh plug from a prior hernia repair had migrated into the bladder. The mesh plug was excised using the Da Vinci Si robot, which allowed for efficient mobilization of the bladder and other anatomic structures, as well as rapid recovery.

***Conclusion:*** Our case demonstrates the need to consider mesh erosion as a cause of hematuria and, furthermore, shows how the robotic approach can help facilitate excision of migrated mesh into the bladder.

## Introduction

Although mesh erosion into adjacent tissues is a well-documented complication of inguinal herniorrhaphy, migration of mesh into the bladder is less common.^1^ Clinical presentation may be misleading as the patient may present with hematuria, thereby prompting a work-up for bladder cancer. Cystoscopy and biopsy may be necessary, as imaging alone cannot always differentiate the two. Our findings emphasize the importance of considering mesh erosion in patients who have the appropriate surgical history and who present with symptoms otherwise suggestive of malignancy, including hematuria. Furthermore, this case exemplifies the ease of use of the four-arm Da Vinci Si robot to allow for mobilization and excision of eroded mesh, as well as the associated benefit of rapid patient recovery.

## Case Presentation

### Clinical history/physical examination

A 62-year-old male presented with painless gross hematuria for 2 years. The patient reported hematuria after activity including exercise and sexual intercourse. Cystoscopy was performed by the referring urologist, which revealed a bladder mass in the left anterior wall of the bladder. Biopsies were obtained by the referring urologist, with equivocal pathology analysis revealing atypia. The patient denied any pelvic pain, flank pain, or dysuria. He denied any unintentional weight loss, fevers, chills, shortness of breath, chest pain, neurologic deficits, headaches, or gastrointestinal disturbances. The patient was eventually referred to us for further evaluation and definitive treatment of the bladder mass.

**FIG. 1.**
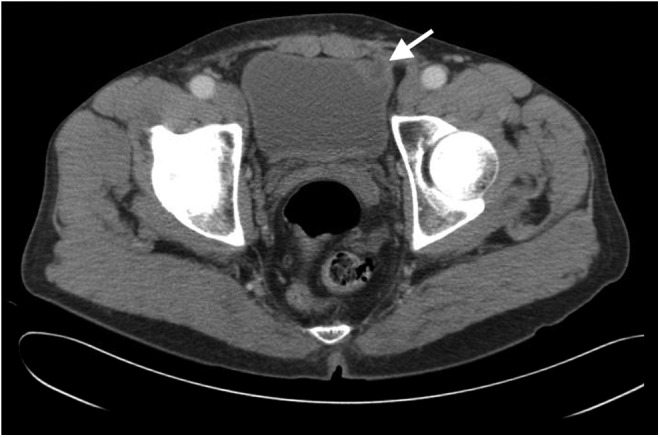
CT of the abdomen with evidence of abnormality at left anterior bladder wall indicated by *white arrow*.

**FIG. 2.**
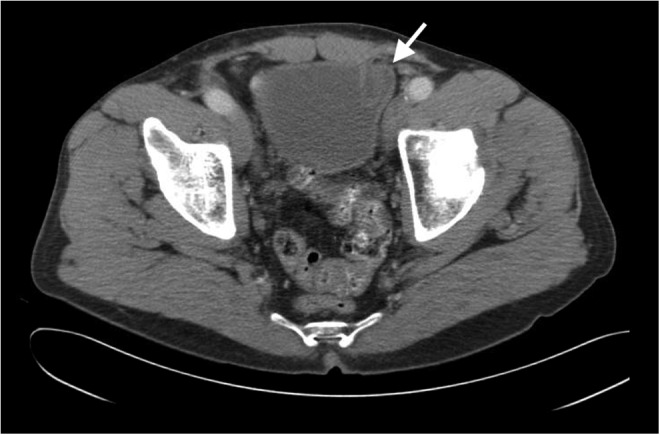
Second image of CT abdomen showing maximal extent of bladder lesion indicated by *white arrow*.

The patient had no significant medical history, except remote history of left inguinal hernia surgery at 3 months of age and a left open inguinal hernia repair with mesh at age 47. There were no prior operative reports available to determine which type of mesh had been used for these repairs. There were no pertinent findings on physical examination. All laboratories, including a complete metabolic panel and a complete blood count, were within normal limits. The patient had a CT of the abdomen and pelvis that showed a mass on the left anterior bladder wall (Figs. 1 and 2).

### Intervention

**FIG. 3.**
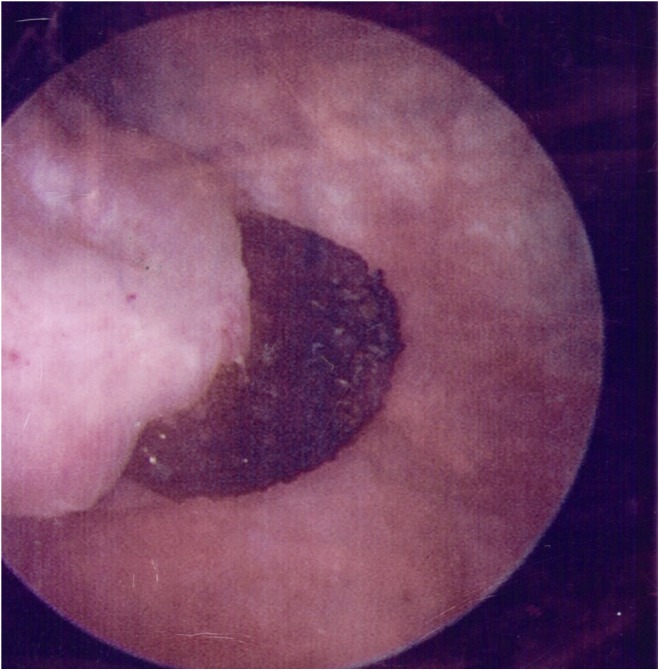
Cystoscopic image of mesh eroding through bladder.

**FIG. 4.**
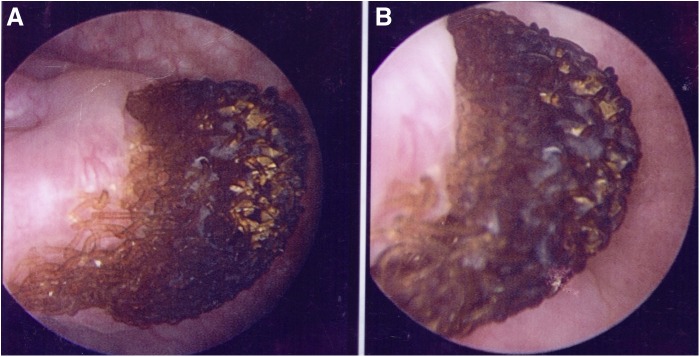
Magnified cystoscopic view showing lattice meshwork of mesh plug.

Given the concern for malignancy and severity of symptoms, the patient ultimately underwent blue light cystoscopy and was found to have a large lesion in the left dome of the bladder. The lesion had a blackened appearance and was found to be blue light negative (Figs. 3 and 4). Closer inspection of the lesion revealed the lattice meshwork, suggestive of an eroded mesh plug. Multiple biopsies were taken to rule out any associated malignancy. The final pathology analysis revealed surgical material consistent with the mesh, as well as benign urothelial mucosa with squamous metaplasia.

We discussed the treatment options with the patient, including the rationale for excision, and the patient opted to proceed with surgery. Surgical excision of the mesh plug was achieved using the robot-assisted laparoscopic approach with the use of the four-arm Da Vinci Si robot. Port placement was similar to transperitoneal robot-assisted radical prostatectomy. A total of five ports were used. A 16F Foley catheter was placed at the beginning of the case. Initially, the sigmoid colon was mobilized out of the pelvis. The bladder was then dropped from the anterior abdominal wall in the usual manner. The mesh plug was transected from the abdominal wall so that the bulk of the mesh remained over the bladder. The bladder was then filled and a partial cystectomy was performed to remove the entirety of the mesh plug, excising a 1-cm margin around the plug. The specimen was removed in an EndoCatch™ bag and the bladder was closed in two layers using absorbable sutures. A 19F Blake drain was placed at the end of the case.

### Follow-up

The postoperative course was unremarkable and the patient was discharged on postoperative day 1. A cystogram was obtained on postoperative day 5 and there was no evidence of contrast extravasation and the foley catheter was removed without difficulty. The final pathology analysis again confirmed mesh fibers with chronic fibrosis and inflammation of bladder mucosa.

## Discussion and Literature Review

Mesh migration into the bladder is a rare complication of inguinal herniorrhaphy.^1^ The presentation is similar to malignancy and may include symptoms of painless hematuria, irritative voiding symptoms, recurrent UTIs or lower pelvic pain.^2,3^ Imaging, such as ultrasonography, may reveal a filling defect or abnormal echogenic mass depending on the depth of involvement of the mesh into the bladder.^2^ CT may reveal a mass lesion within the bladder, but it may not determine whether it is malignant or benign. Even cystoscopy alone may not be enough for a conclusive diagnosis as early mesh migration may present as nonspecific inflammation, papillary projections, or edema.^2^ Biopsy can confirm the absence of malignancy, and if correlated with patient's surgical history, it will allow for a definitive diagnosis.

The pathophysiology of mesh migration is unclear; however, multiple mechanisms may play a role. It has been postulated that mesh migration happens because of movement toward the path of least resistance. This movement occurs because of poor securement of mesh or dislocation caused by external forces.^2,4^ Other theories suggest that mesh migration occurs through anatomic channels and is influenced by erosion caused by a foreign body reaction.^2,4^ Given our patient's surgical history, these mechanisms may have definitely been crucial in mesh migration because of a weak floor with a path of low resistance and excessive inflammation or erosion from multiple repairs. Although the risk is low, hernia recurrence in adulthood in patients who underwent pediatric repair has been quoted to be around 3%.^5^

To the best of our knowledge, there is no clear evidence of inguinal hernia recurrence rates after removal of migrated mesh; however, there is limited data that suggest recurrence rates after removal for mesh infection is lower than 5%.^6^ Regardless of infection or migration, both circumstances require mesh removal, therefore, the recurrence rates may be similar. Nevertheless, more follow-up is needed to evaluate recurrence rates after removal of migrated mesh.

Our case highlights the importance of a complete work-up of any patient with hematuria and suggests that the physician's differential diagnosis should expand if the patient has a history of hernia repair with mesh, especially if the mass lesion within the bladder has an unusual appearance. In addition, the use of the Da Vinci robot allows for easy identification of pertinent anatomic structures and the ability to mobilize the mesh off the abdominal wall efficiently and safely. With this minimally invasive approach, the patient will experience a rapid postoperative recovery with minimal pain and complications.

## Abbreviation Used

CT: computed tomography
